# Bullying and Substance Use in Early Adolescence: Investigating the Longitudinal and Reciprocal Effects Over 3 Years Using the Random Intercept Cross-Lagged Panel Model

**DOI:** 10.3389/fpsyg.2020.571943

**Published:** 2020-11-05

**Authors:** Chiara Filipponi, Serena Petrocchi, Anne-Linda Camerini

**Affiliations:** ^1^Faculty of Communication, Culture and Society, Institute of Communication and Health, Università della Svizzera Italiana, Lugano, Switzerland; ^2^Faculty of Communication, Culture and Society, Institute of Public Health, Università della Svizzera Italiana, Lugano, Switzerland

**Keywords:** bullying, substance use, random intercept cross-lagged panel model, longitudinal study, adolescence

## Abstract

Bullying and substance use among adolescents have been increasingly studied in the field of developmental psychology, but research to date has primarily investigated the cross-sectional relationship and, to a lesser extent, the long-term impact of bullying on substance use. Grounded in the General Theory of Crime, this study focused on the longitudinal reciprocal relationships between bullying and substance use (i.e., smoking and alcohol consumption) during early to mid-adolescence, which is a critical developmental phase. We aimed to investigate the hypothesis of a reinforcing downward spiral at the within-person level. Moreover, we intended to examine gender differences in the expected longitudinal relationships. Our analyses are based on self-report data for bullying and substance use collected from 1,495 adolescents (746 males; *M*_age at T__1_ = 12.42, *SD* = 0.58) at three waves between 2017 and 2019. We applied the random intercept cross-lagged panel model to separate within-person from between-person effects. At the between-person level, the mutual association between bullying and substance use, previously demonstrated in cross-sectional studies, was confirmed. At the within-person level, results provide evidence of a significant age-dependent change in bullying and substance use from 13 to 14 years old, where the significant increase in bullying could be attributed to females but not to males. We also found a gender-independent significant positive effect of bullying at 12 years old on substance use at 13 years, but not vice versa. Thus, the hypothesis of a reinforcing downward spiral, shown by significant positive reciprocal effects, did not find support. According to the General Theory of Crime, our findings underline that bullying can be considered a context-related factor inasmuch as it pushes adolescents to smoke and drink, which are both expressions of low levels of self-control, which need to be considered in intervention programs to effectively prevent unhealthy and risky behaviors in adolescence.

## Introduction

Adolescence is a developmental period characterized by substantial changes in the body and brain (Siegel, 2014). These changes go along with the emergence of conflictual situations as adolescents become increasingly independent from their parents and seek approval by their peers (McLaughlin and Clarke, 2010). This process is oftentimes accompanied by risk-taking and externalizing problem behaviors, including bullying and substance use (Valdebenito et al., 2015). Bullying is a form of goal-directed behavior that involves the repeated exposure to negative actions, with a power imbalance between the bully and the victim (Olweus, 2013; Volk et al., 2014). Bullying can happen not only directly through physical (e.g., punching or slapping, kicking, hitting) and verbal (e.g., name calling, threat) aggressions but also indirectly through relational aggression (e.g., social exclusion) (Sharp and Smith, 2002). Bullying behaviors begin in preschool years (Rigby, 2002), escalate during early adolescence (approximately before the age of 14), and decrease during mid- to late adolescence (approximately after the age of 14) (Pellegrini and Bartini, 2000; Pellegrini and Long, 2002; Rigby, 2002; Inchley et al., 2020). The prevalence of bullying varies according to the study design and context. In their meta-analysis, Modecki et al. (2014) found a weighted average prevalence rate of traditional bullying across 80 studies of about 35%. The latest international report with 2017 and 2018 findings from the Health Behavior in School-aged Children study (Inchley et al., 2020) showed that the proportion of adolescents who reported bullying others ranged from 0.3% among 11-year-old girls (in Portugal) to 30% among 15-year-old boys (in Lithuania). Previous studies showed that the repeated engagement in bullying led to higher rates of mental health problems (Gibb et al., 2011), antisocial and illegal actions (Kim et al., 2011; Ttofi et al., 2011; Wolke et al., 2013), and suicidal behaviors in adulthood (Klomek et al., 2010).

Yet, bullying is not the only externalizing problem behavior during adolescence. Another common health-risk behavior is substance use, which includes cigarette smoking, alcohol consumption, and other forms of illicit drug use (e.g., cannabis, ecstasy, cocaine). Several studies (Radliff et al., 2012; Durand et al., 2013) indicated that substance use normally begins in adolescence, with cigarette smoking and alcohol consumption as the gateway to other forms of illicit drug use in subsequent years (Ren and Lotfipour, 2019). The Health Behavior in School-aged Children study on substance use (Inchley et al., 2020) reported a prevalence of alcohol consumption of 20% in boys and 18% in girls and a prevalence of cigarette smoking of 7% in both genders, when assessed for the last 30 days. The early onset and the repeated consumption of substances can lead to an increased risk of developing addictions (Hingson et al., 2006), subsequent substance-related problems, such as alcohol dependence and substance use disorders (Brook et al., 2002; Magid and Moreland, 2014), and psychiatric disorders (Brook et al., 2002).

Bullying and substance use are oftentimes inter-related. In their systematic review and meta-analysis of 13 cross-sectional studies, Valdebenito et al. (2015) confirmed a strong association between bullying perpetration and substance use. Despite scientific evidence of the cross-sectional relationship between the two behaviors, little is known about their relationship over time. The few longitudinal studies conducted to date showed that early life adversity, such as bullying during elementary school, increased the likelihood of substance use in adulthood (Kim et al., 2011; Niemelä et al., 2011; Stanis and Andersen, 2014). A similar association was found in a retrospective study on young adults (Belacchi, 2009).

Yet, there is still no research to date that examined the *longitudinal reciprocal* associations between substance use and bullying during adolescence. The present paper aimed to fill this gap by drawing on longitudinal data collected over the course of 4 years in early adolescence (i.e., 12–14 years old) (American Academy of Pediatrics [AAP], 2020). The rationale for studying the reciprocal relations between bullying and substance use is guided by theoretical insights from the General Theory of Crime (Gottfredson and Hirschi, 1990).

### General Theory of Crime

Gottfredson and Hirschi (1990) General Theory of Crime posits that low self-control is part of individuals’ personality that takes shape during childhood and adolescence and is responsible for aggressive and impulsive behaviors, sensation seeking, and, ultimately, severe deviant behaviors and crime. Individuals with low self-control tend to be insensitive and self-centered, i.e., less able to identify others’ needs, feelings, and perspectives (Gramick et al., 1993). They prefer immediate gratifications and are less inclined to consider the long-term consequences of their behaviors (Gottfredson and Hirschi, 1990). Studies have shown that aggressive behaviors, such as bullying perpetration, were associated with both a low capability of self-control (Unnever and Cornell, 2003; Chui and Chan, 2013; Fanti and Kimonis, 2013; Moon and Alarid, 2015) and, at the same time, a strong need to control others (Winstok, 2009). Similarly, Wills and Stoolmiller (2002) demonstrated that poor self-control in early childhood determined the escalation of substance use.

Moreover, the General Theory of Crime posits that deviant actions are more likely to occur when a person with low self-control has the opportunity of misconduct (Gottfredson and Hirschi, 1990; Seipel and Eifler, 2010). Nonetheless, they did not specify how this context-related factor should be operationalized and what are the relationships with self-control (Seipel and Eifler, 2010). In this vein, bullies may engage in substance use as a way to gain social status and to be perceived as “cool and attractive” (Spijkerman et al., 2005) in front of other deviant peers (Cook et al., 2010). During adolescence, cognitive control is particularly low (Siegel, 2014), and past studies suggested that adolescents preferred immediate gratifications compared with larger but later rewards (Casey and Jones, 2010). For these reasons, bullying perpetration may represent an opportunity factor for bullies, who have low self-control, to engage in substance use during out-of-school activities. On the other hand, early substance misuse may be a significant predictor of bullying. Having an impact on the neurobiological system of reward and control (Casey and Jones, 2010), substance use decreases the individual level of control and inhibition and, thus, increases the probability of aggressive behaviors.

### Gender Differences

Gender differences have been revealed for both bullying perpetration and substance use. Studies have shown that boys are more likely to be involved in bullying than girls (Nansel et al., 2001; Beaty and Alexeyev, 2008; Cook et al., 2010; Inchley et al., 2020), although girls are more likely to be engaged in indirect relational aggression (Card et al., 2008). With regard to substance use, Quinn et al. (2016) found that, compared with males, females were less likely to smoke, but they had a higher probability of alcohol use. Males showed higher rates of illicit drug use than females (Johnston et al., 2019), and they reported higher rates of substance use as they grew older (White and Bariola, 2012; Johnston et al., 2019).

Gender differences have also been considered in cross-sectional research evaluating the co-occurrence of bullying and substance use and in the longitudinal association between bullying and substance use. Luk et al. (2012) applied latent class analysis on data from adolescents with a mean of age of 14.2 (range 11–15 years old). They found that females were more likely to be in the class of substance users but were less likely to be included in the class of bullies and substance-users-bullies than their male counterparts. In another study, Kim et al. (2011) found no differences in gender when studying the prospective association between bullying behaviors at age 11 and substance use at age 21.

### Study Aim

Considering the above-mentioned gaps in the literature, the aim of the present study was to examine the longitudinal reciprocal relationships between bullying and substance use in adolescence applying a three-wave random intercept cross-lagged panel model (RI-CLPM) (Hamaker et al., 2015). This model allows to separate the within-person effects (i.e., whether the individual-level change in one variable is related to the individual-level change in another variable) from between-person effects (i.e., whether the group-level change in one variable is related to the group-level change in another variable) in order to understand the developmental processes at the intra-individual level.

Based on past studies, we expected to find an increase of bullying and substance use as adolescents grew older (i.e., significant positive lagged effects). Furthermore, we expected to find a reinforcing downward spiral, thus longitudinal interrelations between the two deviant behaviors (i.e., significant positive cross-lagged effects). Given the first evidence of gender differences in bullying and substance use during adolescence, but the lack of evidence regarding the longitudinal reciprocal relationships within males and females, we formulated a research question to examine the possible gender differences in the expected lagged and cross-lagged effects.

## Materials and Methods

### Data Collection

The current study used data from waves 4 (2017), 5 (2018), and 6 (2019) (following T1, T2, and T3) of an ongoing cohort study^1^, which started in 2013 when all public elementary schools in Canton Ticino, Switzerland were invited to participate in the larger cohort study. Based on this opt-in technique, 39 out of 79 schools agreed to participate. Within these schools, a total of 60 grade 4 classes composed of 1,083 students were randomly selected. Parents received a letter on the purpose and the nature of the study, assuring anonymity and confidentiality of all collected data. Each year, teachers received a paper-and-pencil questionnaire for all students in the sample signed with a unique student identifier provided by the Cantonal education administration, instructions for administration, and a pre-stamped return envelope. Teachers accessed the database of the Cantonal education administration to extract the corresponding student names and distribute the questionnaire for self-administered completion at school. They collected all completed questionnaires and sent them back to the research team. Study participation was voluntary, i.e., students were free to leave the questionnaire blank, each year. At wave 3 of the cohort study, when students entered middle school, the cohort was distributed across all 35 public middle schools and 2 private middle schools in Canton Ticino. Resampling was done at class level in underrepresented schools, and randomly selected students and their parents were again informed in writing about the study. Since the use of an identifier assured anonymity of all data and thereby sufficiently addressed ethical considerations regarding privacy, the Cantonal education administration approved the study design.

### Participants

At T1, the initial sample included 1,427 grade 7 students (approximately 12 years old). They were recruited in all 35 public and 2 private middle schools in Canton Ticino, Switzerland. The sample was composed of 1,361 participants at T2 (5% attrition rate from T1 to T2) and 1,224 participants at T3 (14% attrition rate from T1 to T3). Sample attrition was mainly due to students being absent from school at the day of data collection, repeating a school year, or moving away from Canton. Since we kept missing data points when matching the data for all three waves, the analytical sample included 1,495 adolescents. Based on data collected at T1, the sample consisted of 746 males (50%) and had an average age of 12 years (*M*_age_ = 12.42, *SD* = 0.58). The majority (66%) reported a good or very good socio-economic status (range from 0 “not at all good” to 4 “very good”; *M* = 2.84, *SD* = 0.77). Mann–Whitney *U* and χ^2^ tests were conducted to compare participants at T1 to drop-outs at T2. They showed the same distribution of gender [χ^2^(1) = 0.006, *p* = 0.94], perceived socio-economic status (*U* = 29,115, *p* = 0.20), bullying (*U* = 31,269, *p* = 0.82), and substance use (*U* = 29,457, *p* = 0.17). Since the analytical sample made up almost 50% of the entire cohort born in 2004/05 and distributed across Canton (USTAT and UFFICIO Di Statistica, 2020), and no systematic drop-out was evident, the analytical sample can be considered representative.

### Measures

#### Bullying

Five items were used to assess how many times participants engaged in physical, verbal, and relational bullying behaviors during the past 3 months (Mössle, 2012). Items included: “I punched a classmate,” “I made fun of a classmate or said bad things about him,” “I ignored a classmate on purpose,” “I said things about a classmate which were not true,” and “I forced a classmate to do things that he did not want to.” Response options ranged from 1 “never” to 4 “always.” Confirmatory factor analyses (CFAs) confirmed the expected one-factor structure at all three-time points (for details, see Table 1). A final score was created for each wave by calculating the average of the five items, with higher scores indicating higher levels of bullying perpetration (for descriptive statistics and Cronbach’s alpha, see Table 2).

**TABLE 1:** Summary of the fit indices for the CFAs of the bullying scales at the three-time point.

CFA	χ^2^ (df)	CFI	RMSEA (90% CI)	SRMR
Model 1—T1	42.67 (5)***	0.958	0.074 (0.060–0.088)	0.034
Model 2—T2	30.40 (5)***	0.974	0.064 (0.050–0.078)	0.030
Model 3—T3	5.63 (5)^+^	0.999	0.010 (0.00–0.030)	0.013

***p < 0.001. ^+^Non-significant, p = 0.344.

**TABLE 2:** Descriptive statistics of the scales at the three measurement points and results from the reliability analysis.

	M (SD; range)	Skewness (SE)	Kurtosis (SE)	α/r_s_ (rs)
Bullying T1	0.33 (0.47; 0–3)	2.57 (0.06)	8.89 (0.13)	0.80 (>0.56)
M	0.40 (0.48)			
F	0.24 (0.36)			
Bullying T2	0.29 (0.47; 0–3)	2.62 (0.07)	8.78 (0.14)	0.83 (>0.57)
M	0.38 (0.55)			
F	0.21 (0.36)			
Bullying T3	0.29 (0.53; 0–3)	2.94 (0.07)	9.92 (0.14)	0.87 (>0.61)
M	0.38 (0.55)			
F	0.19 (0.36)			
Substance use T1	1.18 (0.52; 1–5)	3.83 (0.06)	16.99 (0.13)	*r*_s_ = 0.40**
M	1.18 (0.49)			
F	1.13 (0.46)			
Substance use T2	1.42 (0.84; 1–5)	2.36 (0.07)	5.22 (0.14)	*r*_s_ = 0.59**
M	1.45 (0.85)			
F	1.33 (0.77)			
Substance use T3	1.65 (1.02; 1–5)	1.64 (0.07)	1.85 (0.14)	*r*_s_ = 0.65**
M	1.72 (1.07)			
F	1.57 (0.96)			
Social desirability T1	3.52 (0.76; 1–5)	-0.27 (0.06)	0.01 (0.13)	0.65 (>0.20)

M, males; F, females; SE, standard error; rs, inter-item correlation; r_s_, Spearman’s rho. **p < 0.01.

#### Substance Use

Two items were used to measure adolescents’ smoking and drinking behaviors during the past 3 months: “I smoked cigarettes (more than a pull)” and “I drank alcohol (more than a shot).” Response options ranged from 1 “never” to 5 “always.” A final score was created by calculating the mean of the two items, with higher scores indicating higher levels of substance use (for descriptive statistics and correlations between the two items, see Table 2).

#### Social Desirability

Given that past research found evidence for a significant social desirability bias when reporting socially unacceptable behaviors, such as bullying and substance use, five items from the Italian version of the Children’s Social Desirability Short Scale were included in the analyses (Camerini and Schulz, 2018). Eight items of the scale were not included because they conceptually overlapped with the bullying scale and created multicollinearity (e.g., “Have you ever felt like saying unkind things to a person?” or “Do you sometimes feel like making fun of other people?”). Response options ranged from 1 “always” to 5 “never.” A final score was created by calculating the mean of the five item, with higher scores indicating higher levels of social desirability (for descriptive statistics and Cronbach’s alpha, see Table 2).

### Data Analysis Strategy

Preliminary data analyses were conducted in SPSS v.25. First, the data were screened for missing values. Next, univariate distributions (i.e., skewness and kurtosis) were examined. All the subsequent analyses were carried out in R using the Rstudio software v.1.2.5019 and the Lavaan package (Rosseel, 2012). A CFA was used to examine whether the proposed unidimensional factor structure of the bullying scale had a good fit to the data in all three measurement points. According to Byrne (2010), a CFA model can be accepted when the χ^2^-value is non-significant. However, on large samples (400 cases or more), the χ^2^-value is highly likely significant. Therefore, we also considered the following goodness-of-fit indices: comparative fit index (CFI) > 0.90, root mean square error of approximation (RMSEA) = 0.08, and standardized root mean residual (SRMR) < 0.08 (Hu and Bentler, 1999).

Before testing the RI-CLPM, bivariate correlations between the main variables and the covariates (age, gender, socio-economic status, and social desirability) were calculated. Furthermore, controlling for gender, a repeated measures ANOVA was conducted for bullying and substance use to evaluate variations over time. For the RI-CLPM and the multi-group comparison by gender, missing data were handled using the Full Information Maximum Likelihood (FIML) method with a robust estimator, which provides reliable estimates and permits the use of all available data. Non-normality of univariate distributions was handled with the maximum likelihood estimation (MLR) with robust (Huber–White) standard errors and a scaled test statistic that is equal to the Yuan–Bentler test statistic (Lai, 2018) and performed well with large sample sizes (Hox et al., 2010).

To test the RI-CLPM, we followed the suggested procedure by Hamaker et al. (2015) comparing the unconstrained model (Model 1) against models in which the autoregressive (Model 2) and cross-lagged path coefficients (Model 3) were constrained to be equal over time. If two models fitted the data equally well, the most parsimonious (i.e., the model with less constraints) was chosen. If adding equality constraints deteriorates model fit, we can conclude that the effects from T1 to T2 are not equal to the effects from T2 to T3, and the within-person associations between bullying and substance use differ as participants grow older. If model fit does not deteriorate, the effects can be considered similar for each time interval and, thus, age independent. The RI-CLPM was tested controlling for general trait-like social desirability measured at T1. Model fit was evaluated using the recommended fit indices and thresholds mentioned above. The Satorra–Bentler-scaled χ^2^-difference test was used to compare model fit (Satorra and Bentler, 2001). For model comparison, the Δχ^2^ was calculated, including ΔBIC and ΔAIC.

Eventually, we performed a multi-group analysis by gender and tested measurement invariance, which is usually tested through three models (Vandenberg and Lance, 2000): configural, weak (i.e., metric invariance), and strong invariance (i.e., scalar invariance). The configural model tests whether the items measure the same construct across years. The weak model tests whether the factor loadings of the items (i.e., the degree to which differences among participants’ responses to the item arise from differences among their levels of the underlying construct) are invariant across years. Finally, the strong model states whether intercepts and errors are equivalent across years.

## Results

### Preliminary Results

Missing data did not exceed the threshold of 10%. Descriptive statistics for the compound scales across the three measurement points indicated distributions with a high kurtosis for both bullying and substance use, especially at T1 and T2 (see Table 1). This is not surprising given the relatively low prevalence rates of these two behaviors in early adolescence, though a small proportion already started to engage in these behaviors on a more frequent basis.

The prevalence rate for bullying ranged from 8.4% at T1 to 7.8% at T3, whereas for substance use, the prevalence rate ranged from 8.2% at T1 to 24.2% at T3. Spearman’s bivariate correlations (Table 3) revealed significant positive relations between bullying and substance use at all three measurement points with coefficients ranging from 0.13 (substance use at T1 with bullying at T3) to 0.37 (substance use at T2 with bullying at T3).

**TABLE 3:** Spearman’s bivariate correlations between variables.

	1	2	3	4	5	6	7	8	9	10
1 Gender (female)	–									
2 Age T1	-0.05*	–								
3 Economic well-being T1	0.02	–0.03	–							
4 Bullying T1	–0.19*	0.05	–0.05	–						
5 Bullying T2	–0.16**	0.06*	0.001	0.32**	–					
6 Bullying T3	–0.17**	0.05	–0.005	0.28**	0.34**	–				
7 Substance use T1	–0.12**	0.10**	–0.06*	0.32**	0.21**	0.15**	–			
8 Substance use T2	–0.12**	0.06**	–0.07*	0.26**	0.36**	0.21**	0.36**	–		
9 Substance use T3	–0.09**	0.08**	–0.06	0.26**	0.22**	0.34**	0.32**	0.43**	–	
10 Social desirability T1	0.14**	–0.04	0.12**	–0.42**	–0.26**	–0.23**	–0.31**	–0.24**	–0.25**	–

*p < 0.05, **p < 0.01.

CFA confirmed the expected one-factor structure for bullying at all three-time points (see Table 3).

The repeated measures ANOVA showed that bullying slightly decreased over time, but the change was not significant. On the other hand, substance use significantly increased over time [*F*(2, 1.88) = 156.87, *p* < 0.001] (see also Table 2 for means and standard deviations). The trend was linear from T1 to T2 [*F*(1, 1049) = 99.99, *p* < 0.001] and from T1 to T3 [*F*(1, 1049) = 274.90, *p* < 0.001]. Furthermore, there was a significant effect of gender for bullying [*F*(1, 1035) = 68.63, *p* < 0.001] and for substance use [*F*(1, 1049) = 8.83, *p* < 0.001] across the three-time points. On average, males reported higher levels of bullying and substance use than females.

### Random Intercept Cross-Lagged Panel Analysis

**FIGURE 1:**
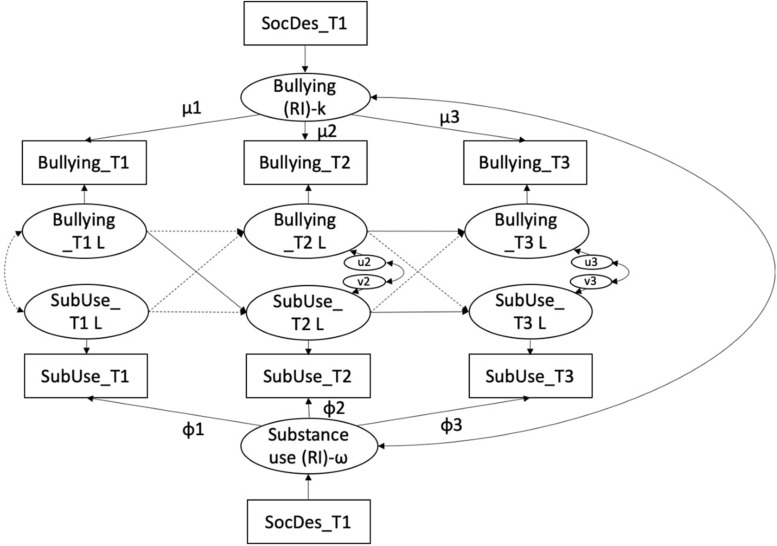
Path diagram of the final unconstrained RI-CLP model. All factor loadings are constrained to 1. RI, Random Intercept; k, Kappa; ω, Omega; μ, mu; φ, phi; L-suffix, Latent factor of the indicator; SubUse, Substance Use; SocDes, Social Desirability. Plain arrows indicate significant paths; dotted arrows indicate non-significant paths.

First, a RI-CLPM was estimated without any constraints (Model 1) but controlling for social desirability bias. Of the analytical sample of 1,495, 95 patterns were missing and, therefore, excluded when estimating the parameters. Model 1 had an acceptable fit to the data: χ^2^(5) = 57.675, *p* < 0.001, CFI = 0.97, RMSEA = 0.07 (90LO = 0.06, 90HI = 0.09, PCLOSE = 0.01), SRMR = 0.04 (see also Table 4). Figure 1 reports a graphical representation of the paths for the entire sample, whereas Table 5 includes a detailed overview of the path coefficients.

**TABLE 4:** Summary of the fit indices for RI-CLPM comparisons and for multi-group invariance analysis by gender.

	χ^2^ (df)	CFI	RMSEA (90% CI)	SRMR	Δχ^2^ (df)	Decision
**RI-CLPM comparison**						
RI-CLPM—Model 1 with no constraints	57.675 (5)	0.968	0.074 (0.057–0.092)	0.040	–	
RI-CLPM—Model 2 with constrained lagged effects	72.354 (7)	0.961	0.064 (0.050–0.078)	0.045	7.1454 (2)*	Rejected (Model 1 vs. Model 2)
RI-CLPM—Model 3 with constrained cross-lagged effects	66.282 (7)	0.963	0.071 (0.056–0.087)	0.046	11.301 (2)*	Rejected (Model 1 vs. Model 3)
**Multi-group invariance analysis by gender**						
Configural invariance (CI)	60.339 (10)	0.968	0.071 (0.054–0.090)	0.041	–	
Weak invariance (WI)	234.843 (28)	0.878	0.070 (0.061–0.079)	0.096	78.158 (18)***	Rejected (CI vs. WI)
Strong invariance (SI)	202.215 (20)	0.895	0.069 (0.060–0.079)	0.079	42.722 (10)***	Rejected (SI vs. CI)

*p < 0.05; ***p < 0.001.

**TABLE 5:** Summary of the parameters for the whole sample and by gender of the unconstrained RI-CLPM (Model 1).

	All (*n* = 1,400)				Females (*n* = 709)				Males (*n* = 691)			
	b	SE	β	p	b	SE	β	p	b	SE	β	p
**Lagged (autoregressive) effects**												
Bullying T1 → bullying T2	0.11	0.08	0.10	0.18	0.10	0.11	0.09	0.39	0.12	0.11	0.11	0.28
Bullying T2 → bullying T3	**0.15**	**0.07**	**0.14**	**<0.05**	**0.27**	**0.11**	**0.26**	**<0.05**	0.06	0.10	0.05	0.52
Substance use T1 → substance use T2	–0.28	0.55	–0.11	0.61	–0.54	1.31	–0.20	0.68	–0.12	0.52	–0.05	0.82
Substance use T2 → substance use T3	**0.46**	**0.07**	**0.36**	**<0.001**	**0.56**	**0.10**	**0.44**	**<0.001**	**0.36**	**0.09**	**0.29**	**<0.001**
**Cross-lagged effects**												
Bullying T1 → substance use T2	**0.33**	**0.14**	**0.18**	**<0.05**	0.47	0.27	0.23	0.08	0.26	0.18	0.15	0.15
Bullying T2 → substance use T3	–0.01	0.10	–0.01	0.91	–0.10	0.17	–0.04	0.56	0.05	0.13	0.03	0.69
Substance use T1 → bullying T2	0.11	0.15	0.07	0.47	0.17	0.24	0.11	0.48	0.13	0.19	0.09	0.48
Substance use T2 → bullying T3	0.07	0.05	0.11	0.13	0.08	0.05	0.15	0.13	0.05	0.07	0.07	0.51
**Covariate effects**												
Social desirability T1 → bullying (Kappa)	**–0.18**	**0.01**	**–0.60**	**<0.001**	**–0.13**	**0.01**	**–0.79**	**<0.001**	**–0.22**	**0.02**	**–0.58**	**<0.001**
Social desirability T1 → substance use (Omega)	**–0.21**	**0.02**	**–0.37**	**<0.001**	**–0.17**	**0.03**	**–0.34**	**<0.001**	**–0.24**	**0.03**	**–0.38**	**<0.001**
**Random intercepts**												
Bullying T1 (mu1)	**0.97**	**0.05**	**2.12**	**<0.001**	**0.71**	**0.06**	**1.93**	**<0.001**	**1.18**	**0.08**	**2.25**	**<0.001**
Bullying T2 (mu2)	**0.95**	**0.05**	**1.94**	**<0.001**	**0.68**	**0.06**	**1.73**	**<0.001**	**1.15**	**0.09**	**2.06**	**<0.001**
Bullying T3 (mu3)	**0.93**	**0.05**	**1.77**	**<0.001**	**0.66**	**0.06**	**1.63**	**<0.001**	**1.15**	**0.09**	**1.84**	**<0.001**
Substance use T1 (phi1)	**1.93**	**0.09**	**3.68**	**<0.001**	**1.77**	**0.11**	**3.78**	**<0.001**	**2.05**	**0.13**	**3.59**	**<0.001**
Substance use T2 (phi2)	**2.19**	**0.09**	**2.58**	**<0.001**	**1.98**	**0.11**	**2.49**	**<0.001**	**2.35**	**0.13**	**2.63**	**<0.001**
Substance use T3 (phi3)	**2.42**	**0.09**	**2.37**	**<0.001**	**2.22**	**0.11**	**2.29**	**<0.001**	**2.59**	**0.13**	**2.42**	**<0.001**
**Covariances—between-person effects**												
Bullying (Kappa)—substance use (Omega)	**0.03**	**0.01**	**0.42**	**<0.01**	**0.01**	**0.01**	**0.38**	**0.44**	**0.05**	**0.02**	**0.47**	**<0.05**
Bullying T1—substance use T1	0.02	0.02	0.18	0.28	0.03	0.02	0.33	0.24	0.01	0.03	0.09	0.61
Bullying T2—substance use T2	**0.13**	**0.02**	**0.42**	**<0.001**	**0.11**	**0.03**	**0.45**	**<0.001**	**0.14**	**0.04**	**0.40**	**<0.001**
Bullying T3—substance use T3	**0.11**	**0.02**	**0.28**	**<0.001**	**0.05**	**0.01**	**0.17**	**<0.001**	**0.18**	**0.04**	**0.37**	**<0.001**

*Results based on n = 1,400 of 1,495 (95 cases excluded due to incomplete patterns). Standardized coefficients and standardized confidence intervals are shown. In bold are shown the significant paths.*

The auto-regressive paths were significant for substance use from T2 to T3 (β = 0.36, *p* < 0.001) and for bullying from T2 to T3 (β = 0.14, *p* < 0.05). The model also included a significant within-person cross-lagged path from bullying at T1 to substance use at T2 (β = 0.18, *p* < 0.05). The between-person correlation between the random intercepts of bullying and substance use was significant (β = 0.42, *p* < 0.001), indicating that individuals who reported higher levels of one risk behavior also reported higher levels of the other behavior. Moreover, we found that social desirability had a negative effect on both random intercepts of the model (β = -0.60, *p* < 0.001 for bullying, β = -0.37, *p* < 0.001 for substance use), meaning that adolescents with a higher tendency to provide socially desirable answers reported lower levels of bullying and substance use.

In a second step, time invariance was tested by constraining autoregressive effects (Model 2) and cross-lagged effects (Model 3). Model 2 with constraints on the lagged effects showed good model fit: χ^2^(7) = 72.354, *p* < 0.001, CFI = 0.96, RMSEA = 0.06 (90LO = 0.05, 90HI = 0.08, PCLOSE = 0.05), SRMR = 0.04. Furthermore, Model 3 with constrained cross-lagged effects showed also good model fit: χ^2^(7) = 66.282, *p* < 0.001, CFI = 0.96, RMSEA = 0.07 (90LO = 0.06, 90HI = 0.09, PCLOSE = 0.05), SRMR = 0.05. Critically, the χ^2^-test difference indicated that Model 2 fit the data worse than the unconstrained Model 1: Δχ^2^(2) = 7.1454, *p* < 0.05. Likewise, the χ^2^-test difference between Model 1 and Model 3 was significant, indicating a deterioration in fit: Δχ^2^(2) = 11.301, *p* < 0.05. For this reason, the most parsimonious model (Model 1) was chosen, meaning that the effects of T1 on T2 were not equal to the effects of T2 on T3 and, thus, depended on adolescents’ age for both the autoregressive and cross-lagged effects.

### Multi-Group Analysis by Gender

In a third and final step, we performed a multi-group analysis by gender of the unconstrained model (Model 1). We tested the baseline model (i.e., configural model) in each group. The configural model had a good fit to the data, χ^2^(10) = 60.34, *p* < 0.001, CFI = 0.97, RMSEA = 0.07 (90LO = 0.05, 90HI = 0.09, PCLOSE = 0.02), SRMR = 0.04. The between-person correlation between the random intercepts of bullying and substance use was significant only for males (β = 0.47, *p* < 0.05), indicating that males–but not females–who reported higher levels of one risk behavior also reported higher levels of the other behavior. However, both males and females showed a significant and negative effect of social desirability on both random intercepts of the model, with values being slightly higher in females for bullying (β = -0.79, *p* < 0.001) and males for substance use (β = -0.38, *p* < 0.001).

At the within-person level, we did not find a significant cross-lagged effects of the two deviant behaviors, but only significant lagged effects that were different in both groups. For males, the model showed a significant auto-regressive path from T2 to T3 for substance use (β = 0.29, *p* < 0.001). For females, the model showed significant lagged effects from T2 to T3 for substance use (β = 0.44, *p* < 0.001) and bullying (β = 0.26, *p* < 0.05).

**FIGURE 2:**
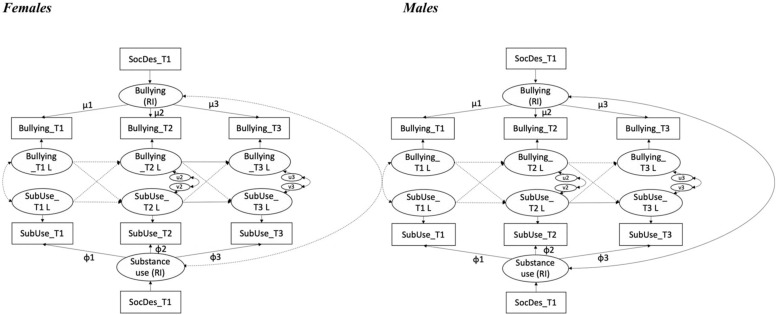
Path diagram of the final RI-CLP Models by gender. All factor loadings are constrained to 1. RI, Random Intercept; k, Kappa; ω, Omega; μ, mu; φ, phi; L-suffix, Latent factor of the indicator; SubUse, Substance Use; SocDes, Social Desirability. Plain arrows indicate significant paths; dotted arrows indicate non-significant paths.

Thus, we performed gender invariance across time points by testing weak and strong invariance. Imposing equality constraints on the model led to a decrease in the fit indices for both the weak and strong invariance models, as shown in Table 4. The calculated Δχ^2^s showed that the configural model was better than the other constrained models, meaning that the paths of the RI-CLPM were not gender invariant. A graphical representation of the models for males and females is shown in Figure 2, whereas Table 5 includes the path coefficients for each model.

## Discussion

Risk behaviors during adolescence have been shown to enhance the likelihood of negative health outcomes in later life (Durand et al., 2013). Thus, researchers have dedicated extensive effort in understanding the development of risk behaviors, including bullying (Olweus, 1997; Ttofi and Farrington, 2010, 2012; Azeredo et al., 2015) and substance use (Swadi, 1999; Weinberg, 2001; Silberg et al., 2003; Cleveland et al., 2008) in adolescence to inform effective prevention programs. Evidence exists that bullying and substance use are interrelated, but this suggestion is primarily based on cross-sectional data (Valdebenito et al., 2015).

The present study aimed to add scientific evidence to the discourse by investigating the longitudinal reciprocal relationships between the two constructs. The focus in our study was on the within-person effects, and how substance use and bullying reinforce each other over time as adolescents grow older. To investigate these effects, we applied the RI-CLPM, which allows to separate within-person from between-person effects and to disentangle the effect of the developmental changes. Three-wave data were used, each collected 1 year apart from a cohort of 1,495 adolescents aged 12 at the time of the first assessment.

Before discussing our results regarding within-person effects, it should be noted that we found that bullying and substance use were positively related to each other at the between-person level, meaning that higher levels of bullying perpetration were associated with higher levels of substance use and *vice versa*. This result confirms previous findings about the co-occurrence of the two deviant behaviors during adolescence, as evidenced in a meta-analysis on cross-sectional research (Valdebenito et al., 2015).

For what concerns the results for our main research objectives, i.e., the examination of the longitudinal reciprocal effects of the two behaviors at the within-person level, we found, first of all, an increase of self-reported bullying behaviors and substance use toward the end of early adolescence, i.e., when our participants were 13 and 14 years old, respectively. This evidence partly supports our initial hypothesis on an increment of the two behaviors across all three-time points, and it confirms previous findings on the increment of bullying (Pellegrini and Bartini, 2000; Pellegrini and Long, 2002; Rigby, 2002) and substance use (Radliff et al., 2012; Durand et al., 2013) during this developmental stage. Our findings are particularly informative considering that we controlled for social desirability bias in the analyses. This has been rarely done in studies with younger populations and on sensitive topics, such as bullying and substance use, which generally have a negative connotation and potentially push adolescents to underreport such behaviors to present themselves in the best possible light (Camerini and Schulz, 2018).

Besides an increase of bullying and substance use toward the end of early adolescence, we found that, at the within-person level, early bullying perpetration at T1 predicted substance use at T2, which, in turn, predicted substance use at T3. Thus, bullying can be considered a longitudinal risk factor of substance use, but not *vice versa*, which provides only partial support for our hypothesis on the reinforcing spiral. Furthermore, the results are age-dependent, i.e., specific to the period between 12 and 14 years old. Hence, we can conclude that adolescence is an unstable developmental period, in which many biological, cognitive, and social changes pervade the individual (Siegel, 2014). Due to the many changes during this developmental period, adolescents are particularly susceptible to at-risk behaviors, which are likely to be a consequence of low-self-control (Wills and Dishion, 2004; Casey and Jones, 2010). From the biological changes perspective, Siegel (2014) explained that, during adolescence, the prefrontal cortex in the brain gets re-organized and reshaped. According to the General Theory of Crime (Gottfredson and Hirschi, 1990), this process is influenced by context-related factors, where bullying can be considered a context-related factor inasmuch as it pushes adolescents to smoke and drink, especially when adolescents exhibit low levels of self-control (Unnever and Cornell, 2003; Chui and Chan, 2013; Fanti and Kimonis, 2013; Moon and Alarid, 2015). More precisely, bullies may use substances as a way to gain social status and to be perceived as “cool and attractive” (Spijkerman et al., 2005), especially when in company with other deviant peers (Cook et al., 2010).

The opposite, that is the fact that substance use may lead to an increase in bullying behaviors or a reinforcing spiral, could not be demonstrated in our study. This can be explained by the typically different onsets of the two behaviors. According to Durand et al. (2013), (school) bullying has been shown to begin early in adolescence or even in childhood, whereas the onset of substance use happens in middle and late adolescence. Another explanation may be that, in our study, the causal link between early substance use and later bullying behaviors was masked because we did not consider different susceptibility factors at the personal level (e.g., personality traits, self- and emotional control, moral disengagement, attachment) and social level (e.g., parental monitoring, perceived social support, perceived pressure from peers). Thus, future studies should consider these differential susceptibility factors to provide further evidence on the within-person longitudinal bidirectional relationships between substance use and bullying.

In the present study, we considered gender as a differential susceptibility factor because past research pointed toward gender differences in bullying and substance use during adolescence. We had no *a priori* hypothesis yet formulated a research question to guide our test whether the longitudinal reciprocal relationships between bullying and substance use were gender invariant. At the between-person level, we found a significant correlation between the random intercepts of the two deviant behaviors only for males, demonstrating that those who reported higher levels of bullying also reported higher level of substance use. Luk et al. (2012) came to a similar conclusion, with a different study design, showing a higher co-occurrence of the two deviant behaviors in males than in females. Thanks to our analyses separating between-person from within-person effects, we found no significant cross-lagged effect between bullying and substance use, neither in females nor in males. The only significant results pertain to lagged effects. While in females both substance use and bullying significantly increased from the age of 13–14, in males, only substance use increased during the same period, probably because males already reported higher levels of bullying at the first time of assessment.

This study has several limitations that should be addressed. First, all the measures considered in the present study were based on self-report. Although the model controlled for social desirability bias, other biases, such as recall, and estimation bias remain a potential threat to construct validity. Therefore, future studies should consider the possibility to measure bullying and substance use with other forms of measurement (i.e., through observations or peer-report). Second, bullying was measured with a multi-item scale tapping into different dimensions of the behavior (e.g., physical, verbal, and relational). However, the scale was not extensive and robust enough to consider the different behavioral sub-dimensions separately. It would be interesting to investigate the within-person relationships between the more prevalent form of physical bullying and substance use in males as well as verbal and relational bullying and substance use in females. In addition, substance use was assessed with only two indicators (tobacco and alcohol consumption) and, thus, did not cover other potentially critical substances that have become popular in the last few years, such as electronic cigarettes as well as marijuana or synthetic drugs. Third, the data of this study stem from three waves collected over the course of 3 years during early adolescence. As many risk behaviors, including substance use, take off during middle and late adolescence, future research should apply the RI-CLPM capturing the transition from mid- to late adolescence or even longer developmental periods. An extended period may also reveal potential gender differences in the within-person cross-lagged effects between substance use and bullying. Moreover, it is important to take into account other personal (e.g., personality traits, self- and emotional control, moral disengagement, attachment) and social (e.g., parental monitoring, perceived social support, perceived pressure from peers) susceptibility factors in order to expand the study of the interrelations between the two deviant behaviors.

Despite these limitations, the present research provides a valuable contribution to the literature on the longitudinal relationships between substance use and bullying in adolescence, which are likely to affect future development and health outcomes in adulthood. From a theoretical point of view, our findings confirm the importance of self-control and self-regulation in predicting adolescents’ adjustment and health behaviors (Moffitt et al., 2011). In particular, the present research sheds light on bullying as a risk factor of substance use. This risk asset should be cautionary taken into account when studying the vulnerability mechanisms during adolescence (Blum et al., 2002). Future research should further develop this line of research by including personal and social factors influencing both bullying and substance use, such as personality traits, attachment style, peer relationships (Fossati et al., 2012), and theory of mind and trust toward adults and peers (Rotenberg et al., 2015). From a practical point of view, our findings demonstrate that intervention programs should integrate different types of adolescents’ expressions of self- and emotional control because their interdependence could have an impact on the prevention of unhealthy and risky behaviors. For example, school-based bullying prevention programs targeting early adolescents (see also Ttofi and Farrington, 2011; Evans et al., 2014) should not only consider the specific context of bullying and, consequently, limit their aims to reduced bullying and victimization rates but also already address substance use as a related risk behavior. This can be done by identifying the common underlying self- and emotional control mechanisms in early adolescence. Furthermore, prevention programs should take into consideration the different developmental profiles and vulnerabilities of males and females. Gender-specific programs (Guthrie and Flinchbaugh, 2001) provide a good starting point, though they are easier to be implemented as a family-based, peer-based, or online program, and their effectiveness requires further evaluation (Kumpfer et al., 2008).

## Data Availability Statement

The raw data supporting the conclusions of this article will be made available by the authors, without undue reservation.

## Ethics Statement

Ethical review and approval was not required for the study on human participants in accordance with the local legislation and institutional requirements. However, the regional education administration approved this study design. Written informed consent from participants’ legal guardian/next of kin was not required to participate in this study in accordance with the national legislation and the institutional requirements. However, consent was implied via completion of the questionnaire.

## Author Contributions

A-LC provided funding, conceived the project, and collected the data. CF performed the analyses, and together with SP wrote the manuscript. All authors revised and approved the final version of the manuscript.

## Conflict of Interest

The authors declare that the research was conducted in the absence of any commercial or financial relationships that could be construed as a potential conflict of interest.

## Footnotes

1 http://www.mediaticino.usi.ch
